# Transcranial direct current stimulation for patients with walking difficulties caused by cerebral small vessel disease: a randomized controlled study

**DOI:** 10.3389/fnagi.2024.1511287

**Published:** 2025-01-07

**Authors:** Qiaoqiao Xu, Wenwen Yin, Xia Zhou, Shuo Wang, Sishi Chen, Jiajia Yang, Chunhua Xi, Zhongwu Sun

**Affiliations:** ^1^Department of Neurology, The Third Affiliated Hospital of Anhui Medical University (Hefei City First People's Hospital), Hefei, Anhui, China; ^2^Department of Neurology, The First Affiliated Hospital of Anhui Medical University, Hefei, Anhui, China; ^3^Department of Rehabilitation, The First Affiliated Hospital of Anhui Medical University, Hefei, Anhui, China; ^4^Department of Burns and Wound Healing, The First Affiliated Hospital of Anhui Medical University, Hefei, Anhui, China

**Keywords:** cerebral small vessel disease, gait disorders, transcranial direct current stimulation, walking difficulties, cognitive impairment, gait analysis, neurovascular coupling, magnetic resonance imaging

## Abstract

**Introduction:**

Cerebral small vessel disease (CSVD) is a chronic systemic degenerative disease affecting small blood vessels in the brain, leading to cognitive impairments. Transcranial direct current stimulation (tDCS), a non-invasive brain stimulation technique that applies low electrical currents to the scalp, shows promise in treating cognitive and movement disorders. However, further clinical evaluation is required to assess the long-term effects of tDCS on neuroplasticity and gait in patients with CSVD. We investigated the effects of long-term, repeated tDCS on local brain perfusion, network connectivity, cognition, and gait in patients with CSVD and gait disorders (CSVD-GD).

**Methods:**

This prospective, single-blind, multicenter, randomized controlled study enrolled 66 patients with CSVD-GD, categorized into the tDCS and Sham groups. Imaging and gait characteristic data were collected over three periods using magnetic resonance imaging and a gait analyzer, along with neuropsychological assessments.

**Results:**

Among 156 volunteers with CSVD-GD, 66 participated in this study, with 60 completing the entire process. Compared to the Sham group, the tDCS group exhibited a more pronounced increase in the cerebral blood flow to the dural cerebrospinal fluid ratio in regions such as the orbitofrontal cortex and cingulate gyrus (*P* < 0.05, FDR corrected), along with significantly greater improvements in gait speed and stride length. Tolerance to tDCS was good, with no difference in adverse reactions between the groups, except for a scalp burning sensation reported during the 1st week (24.24% and 6.06% in the tDCS and Sham groups, respectively; *P* = 0.003).

**Discussion:**

Long-term tDCS is effective and safe for improving neuroplasticity and gait cognition in patients with CSVD.

## 1 Introduction

Cerebral small vessel disease (CSVD) is a common form of cerebrovascular disease that predominantly affects small arteries and microvessels, leading to cerebral ischemia and hemorrhage. The clinical manifestations of CSVD include cognitive impairments, reduced motor function, and emotional disturbances (Li et al., 2018; Ogama et al., 2021). Changes in gait often reflect a decline in physical activity capacity and can precede cognitive decline, serving as critical indicators for identifying and predicting vascular cognitive impairment and fall risk (Montero-Odasso et al., 2012; Adam et al., 2023). Patients with CSVD exhibit significantly increased gait instability and fall risk, which adversely affect their quality of life and place an additional burden on caregivers. Accordingly, the pursuit of effective therapeutic strategies to improve walking ability in patients with CSVD has emerged as a focal point in clinical research.

In the context of neurological diseases, disorders of postural control and balance frequently show insufficient responses to pharmacotherapy, with treatments often only partially alleviating symptoms or failing to impede disease progression. Consequently, there is a pressing need for alternative interventions. Recent studies indicate that transcranial direct current stimulation (tDCS) can modulate cortical excitability, thereby promoting motor learning and enhancing motor function, providing a promising avenue for rehabilitation in patients with CSVD (Ko, 2021; Sudbrack-Oliveira et al., 2021).

tDCS is a non-invasive technique that applies weak direct currents to the scalp to regulate the excitability of cortical neurons. Its underlying mechanism involves altering the membrane potential of neurons, which influences neural conduction and plasticity. Numerous studies have shown that tDCS can facilitate motor learning and improve functional performance, particularly in patients with neurodegenerative diseases who present both cognitive and motor challenges (Manor et al., 2021; Sorkpor and Ahn, 2021; Veldema and Gharabaghi, 2022; Nombela-Cabrera et al., 2023; Lescrauwaet et al., 2024). However, research on the effects of tDCS within the CSVD population is still in its early stages, and specific outcomes have yet to be comprehensively validated (Niemrungruang et al., 2024).

Long-term tDCS treatment may deliver sustained improvements in walking ability among patients with CSVD. Persistent neural stimulation reportedly enhances neuroplasticity and improves motor function (Alkhasli et al., 2022). Implementing long-term tDCS interventions for patients with CSVD may reverse motor function impairments caused by small vessel pathology. Furthermore, the side effects of tDCS are relatively minimal, rendering it suitable for prolonged use in older patients, providing a safe and effective treatment option for those with CSVD (Ko, 2021).

We hypothesized that the application of tDCS in individuals with CSVD may enhance cerebral plasticity by modulating cerebral blood flow and neurovascular coupling, ultimately improving cognitive function, motor control, and gait performance, thereby enhancing walking ability in these patients. Therefore, in this study, we aimed to explore the cumulative neural effects of repeated stimulation by conducting multiple, long-term, prospective observations using multi-parameter magnetic resonance imaging (MRI) along with assessments of gait and cognitive-behavioral changes. The goal is to investigate further the efficacy of neuromodulation techniques in addressing the complexities of CSVD and enhancing patient quality of life.

## 2 Materials and methods

### 2.1 Ethics

This study was approved by the Anhui Medical University Ethics Committee (ethics number: 83240045), registered under MR-34-24-022386, and performed in accordance with the Declaration of Helsinki.

### 2.2 Study participants

This study included 66 patients with CSVD and gait disturbances. The inclusion criteria were as follows: (1) gait disturbances indicated by Timed Up and Go (TUG) test times >15 s using a semi-quantitative analysis method; (2) the presence of at least one of the following CSVD imaging features (Duering et al., 2023): ≥1 lacunar infarctions, a Fazekas score for periventricular white matter hyperintensity ≥3 or deep white matter hyperintensity ≥2 (Fazekas et al., 1993), cerebral microbleeds of ≥1, and perivascular spaces (PVS) ≥ 11 on one side of the basal ganglia or centrum semiovale; and (3) the ability to complete all examinations. The exclusion criteria were as follows: (1) acute or chronic cerebral hemorrhage, acute cerebral infarction, brain tumors, a history of cranial trauma or neurosurgery; (2) non-vascular white-matter hyperintensities caused by conditions such as multiple sclerosis, toxic encephalopathy, encephalitis, or infection; (3) other diseases that may cause gait disorders, such as Alzheimer's disease, Parkinson's disease, Lewy body dementia, and other diseases causing cognitive impairment (Strubel et al., 2001; He et al., 2024; Lin et al., 2024); (4) severe visual, auditory, or language impairments; (5) the presence of dental or metal implants; (6) systemic organ dysfunction or systemic malignancies; and (7) a history of epilepsy.

Participants were recruited from the Neurology outpatient and inpatient departments of the First Affiliated Hospital of Anhui Medical University and the Third Affiliated Hospital. The PASS 11.0 software was used to assess the required sample size. The sample size was estimated for 80% power and a two-tailed α level of 5% for the CSVD-GD. This study was powered to detect a between-group difference of at least three points, with an estimated attrition rate of 10%. Consequently, a target sample size of 56 patients (28 per group) was established; ultimately, 66 patients (33 per group) were enrolled, approximately 18% higher than the anticipated sample size. This over-recruitment was implemented primarily to enhance the reliability of the study results and to ensure sufficient statistical power in the data analysis.

### 2.3 Experimental design

This study is a randomized, single-blind, multicenter prospective trial. Participants were randomly assigned to either the tDCS group or the Sham group. The detailed randomization process can be found in the Supplementary File. Patients or their family members provided informed consent prior to any experimental procedures, including the collection of general medical history, cognitive function assessments, and imaging examinations.

This study adopted a parallel control design, with active stimulation of the left dorsolateral prefrontal cortex using tDCS (2 mA continuous for 30 min) as the experimental group and a sham condition (2 mA continuous for 30 s followed by no stimulation for 29.5 min) as the control group. Stimulation was administered three times a week for six consecutive weeks to observe therapeutic effects and record any adverse reactions during treatment. The study primarily focused on the cumulative effects of tDCS, with imaging, gait assessments, and neuropsychological scale evaluations conducted at baseline, the 4th week, and the 6th week (study endpoint) following tDCS sessions. The final assessment was performed 24 h after tDCS to eliminate the immediate effects of stimulation. Evaluations were performed by trained neurologists who were blinded to the patient's conditions.

The primary outcome was the cerebral blood flow (CBF)/degree centrality (DC) ratio changes in brain regions over time. Although CBF does not directly measure neural activity, it is closely correlated; fluctuations in neural activity affect CBF and changes in neural activity correspond to variations in metabolism (i.e., resting state activity) (Longden et al., 2017). DC is a graph theory-based analysis method that assesses the sum of significant functional connectivity weights between each voxel and all other voxels in the brain (Xiong et al., 2021). The CBF/DC ratio quantifies blood supply per unit of connection strength, reflecting the degree of neurovascular coupling in specific voxels or regions. Assessing cross-voxel correlations between CBF and DC, as well as CBF/DC ratios, can provide insights into neurovascular coupling in patients with CSVD, thereby elucidating the underlying mechanisms contributing to gait disturbances in this demographic. The secondary outcomes included changes in stride length and walking speed over time.

### 2.4 Transcranial direct current stimulation

Participants were seated comfortably in the laboratory. A pair of electrode pads containing two 5 × 7 cm electrodes soaked in saline delivered a current of 2 mA. The electrodes (anode and cathode) were connected to a continuous current stimulation device (TCT, Nanjing, China). According to the 10–20 EEG International System (Jasper, 1958), the anode electrode was placed on the left dorsolateral prefrontal cortex at the location of the F3 electrode, while the cathode electrode was placed on the right frontal pole cortex at the Fp2 electrode position, including the orbitofrontal cortex (BA 10, 11) (Kringelbach, 2005; Moayedi et al., 2015). Both electrodes were secured with a headband. The two electrodes were positioned longitudinally along the medial axis of the target area. The distance at the edges was maintained at least 6 cm apart to reduce current shunting through the scalp between the electrodes. The stimulation was gradually increased during the initial 30 s and then ramped down during the final 30 s. The ramp-up and ramp-down periods were completed within 30 s, after which the device was turned off to serve as a sham condition. This sham stimulation method has been shown to be reliable (Gandiga et al., 2006). Participants typically reported initial sensations of tingling or itching; the stimulation for both groups lasted 30 min.

### 2.5 Clinical and neuropsychological assessments

Demographic information was collected from all participants. Trained neuropsychological technicians performed tests for all participants, completing each patient's assessment within a day. Global cognitive functions were assessed using the Montreal Cognitive Assessment (MoCA). Executive functions were measured using the Trail Making Test-B (TMT-B) and the Stroop Color-Word Test-C (SCWT-C); visuospatial abilities were examined using the Clock Drawing Task, and processing speed was gauged using TMT-A. Raw scores for each test were standardized using z-transformation based on the mean and standard deviation of the control group. Each domain score was calculated by averaging the *z*-scores from relevant neuropsychological tests.

### 2.6 MRI scanning and processing

Scans were performed using the 3.0 T Discovery MR750w MRI system (GE Healthcare, Chicago, IL, USA), equipped with a 24-channel head coil. Participants were instructed to lie quietly with their eyes closed, avoiding head movement, falling asleep, or engaging in cognitive tasks, such as thinking about their problems. The scanning sequences included three-dimensional (3D) pseudo-continuous arterial spin labeling (ASL), 3D brain volume T1-weighted imaging, T2-weighted fluid-attenuated inversion recovery, and susceptibility-weighted imaging. ASL is a non-invasive MRI technique recognized for its excellent reproducibility in measuring CBF. It is primarily used in research related to cerebrovascular diseases, dementia, and neuro-oncology (Fujima et al., 2020). Detailed sequence parameters and preprocessing steps can be found in the Supplementary File.

### 2.7 CBF data preprocessing and computation process

The CBF data preprocessing comprised the following stages: (1) checking data quality and converting images to NIFTI formats; (2) non-linearly registering the cerebral blood flow images to the positron emission tomography image template provided by the Montreal Neurological Institute and performing image quality checks; (3) data normalization through z-score transformation of CBF values for each voxel by dividing the value by the mean CBF value of the whole brain; (4) performing Gaussian smoothing with a full width at half maximum of 6 mm; (5) correcting multiple comparisons using the false discovery rate (FDR) method and a threshold of *P* < 0.05; (6) non-linearly transforming the results to the anatomical automatic labeling (AAL) template, and presenting the final results using xjview (Beijing, CN) at https://www.alivelearn.net/xjview/ (accessed on 12 August 2023) and BrainNet Viewer (Beijing, CN) at http://www.nitrc.org/projects/bnv/ (accessed on 12 August 2023) software.

### 2.8 fMRI data preprocessing and computation process

Data preprocessing was performed using Statistical Parametric Mapping 12 (SPM12; http://www.fil.ion.ucl.ac.uk/spm) on the MATLAB platform. The resting-state fMRI data processing assistant software (DPARSF, http://rfmri.org/dparsf; DPABI 4.3, http://rfmri.org/dpabi) was utilized for data preprocessing. Detailed sequence parameters and preprocessing steps can be found in the Supplementary File. The time series of each voxel was extracted, and the Pearson correlation coefficient with all other voxels in the brain was calculated using a threshold of 0.25 (Garrison et al., 2015). A lower threshold may include false-positive connections, while a higher threshold may exclude some meaningful connections. Consequently, the correlation coefficients were transformed using the Fisher-Z transformation to improve normality.

### 2.9 Gait measurement and assessment

Gait assessment included qualitative, semi-quantitative, and quantitative evaluations. Qualitative analysis involves clinical assessment, observing gait patterns, and identifying muscle and joint issues. The semi-quantitative assessment utilizes clinical scales, such as the TUG test, TUG dual-task (DTUG) test, and Berg Balance Scale for scoring. Specifically, we recorded step length, walking speed, and step frequency upon completion of the TUG test. To ensure comprehensive measurement, we collected data at different stages of the TUG test, including participants' performance on straight paths and during turning segments. On straight paths, we measured participants' step length and walking speed to assess their basic gait characteristics in the absence of obstacles. Similarly, during the turning segments, we recorded step length and walking speed to evaluate participants' gait performance while changing direction. In the DTUG test, participants identified a series of numbers displayed on a computer screen during which they were required to walk while simultaneously locating and verbally articulating the numbers that contained the digit 7. The time and number of errors were recorded. For quantitative gait assessment, we used the Intelligent Device for Energy Expenditure and Activity (IDEEA; MINISUN Co., Manchester, UK), a portable device known for its high test-retest reliability and validity in recording and analyzing gait data (Gorelick et al., 2009). Quantitative measurements using IDEEA parameters included step length, walking speed, and step frequency.

### 2.10 Statistical analyses

Statistical analysis was performed using SPSS 25.0 (IBM Corp., Armonk, NY, USA). The normality of clinical data, such as general information, was assessed using the Shapiro–Wilk test, with parametric data presented as means ± standard deviations (SD) for normally distributed data and as medians (interquartile ranges) [M(Q25, Q75)] for non-normally distributed data. Categorical data are presented as *n*/%. An independent samples *t-*test was used for normally distributed continuous variables, while the Mann–Whitney test was used for non-normally distributed variables. Repeated measures analysis of variance (ANOVA) was used to analyze data across three-time points. Pearson's chi-square test was used for categorical data.

The CBF/DC ratio for each voxel was calculated to evaluate the blood supply amount per unit connection strength and was converted to *z*-scores for each participant to increase normality. Age, sex, education level, and other factors were considered as covariates. Multiple comparisons were corrected using the FDR method, with a threshold of *P* < 0.05.

In regions showing significant differences between the two groups, CBF/DC ratio data were extracted from regions of interest and analyzed using Pearson's correlation analysis based on gait and neuropsychological scales, The plot was generated using R software (v.4.2.2) package ggplot2 (v.3.4.2).

## 3 Results

### 3.1 Participants

**Figure 1:**
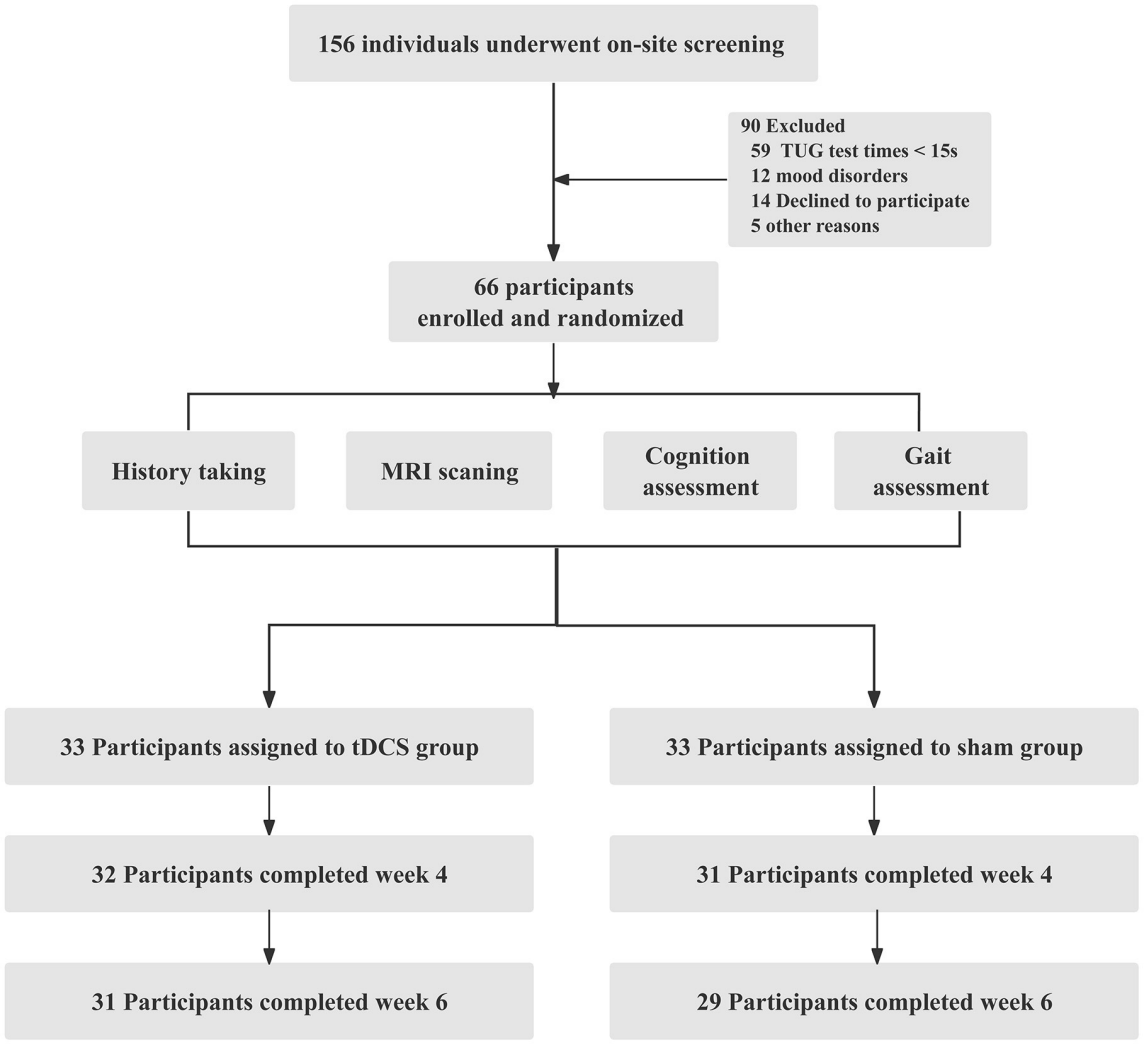
Flowchart depicting the screening process for participant inclusion.

Among the 156 volunteers with CSVD-GD, 66 participated in this study, with 60 completing the entire process. The 66 participants included 25 females with an average age of 68.44 ± 6.16 years and 41 males with an average age of 68.76 ± 6.04 years. The participant enrollment in both groups is illustrated in Figure 1. No statistically significant differences were found between the two groups in terms of general clinical data, gait, and neuropsychological scale scores, including age, sex, and education level (*P* > 0.05, Table 1).

**Table 1:** Patient characteristics.

	**tDCS group (*N* = 33)**	**Sham group (*N* = 33)**	***P*-value**
Age, years	68.24 ± 8.16	67.43 ± 10.54	0.768^b^
Male sex, *n* (%)	22 (20.5)	19 (20.5)	0.447^a^
Education, years	9.36 ± 3.77	7.91 ± 3.88	0.197^b^
BMI, kg/m^2^	23.35 ± 2.80	22.95 ± 3.40	0.658^b^
Current smoking, *n* (%)	13 (11.5)	10 (1.5)	0.438^a^
Hypertension, *n* (%)	14 (10.4)	12 (9.6)	0.614^a^
Diabetes mellitus, *n* (%)	6 (5.5)	5 (5.5)	0.741^a^
Hyperlipemia, *n* (%)	16 (17)	18 (17)	0.622^a^
Total CSVD load (score)	2.00 (1.00, 3.00)	2.00 (1.00, 3.00)	0.190^c^
**Global cognition**			
MoCA (score)	22 (18, 26)	22 (18, 25)	0.506^c^
**Executive**			
TMT-B (s)	176.52 ± 86.37	174.39 ± 96.48	0.936^b^
SCWT-C (s)	47.40 ± 25.66	47.12 ± 26.34	0.971^b^
**Visuospatial**			
CDT (score)	3 (1, 3)	2 (2, 3)	0.883^c^
**Processing speed**			
TMT-A (s)	115.17 ± 60.15	110.85 ± 66.27	0.814^b^
**Gait**			
TUG (s)	16.82 ± 0.25	16.82 ± 0.25	0.698^b^
DTUG (s)	17.82 ± 0.28	19.87 ± 3.32	0.914^b^
BBS (s)	37.92 ± 0.87	37.91 ± 2.75	0.673^b^
Step length (m)	0.48 ± 0.08	0.49 ± 0.08	0.773^b^
Gait speed (m/s)	0.80 ± 0.15	0.84 ± 0.12	0.339^b^
Cadence (spm)	99.95 ± 11.77	99.13 ± 11.81	0.809^b^

^a^Chi-squared test.

^b^Two-sample t-test.

^c^Mann–Whitney test.

tDCS, transcranial direct current stimulation; BMI, Body Mass Index; CSVD, cerebral small vessel disease; MoCA, Montreal Cognitive Assessment; TMT, Trail Making Test; SCWT, Stroop Color-Word Test; CDT, Clock Drawing Test; TUG, Timed Up and Go Test; DTUG, dual-task Timed Up and Go test; BBS, Berg Balance Scale.

### 3.2 Primary outcome

**Figure 2:**
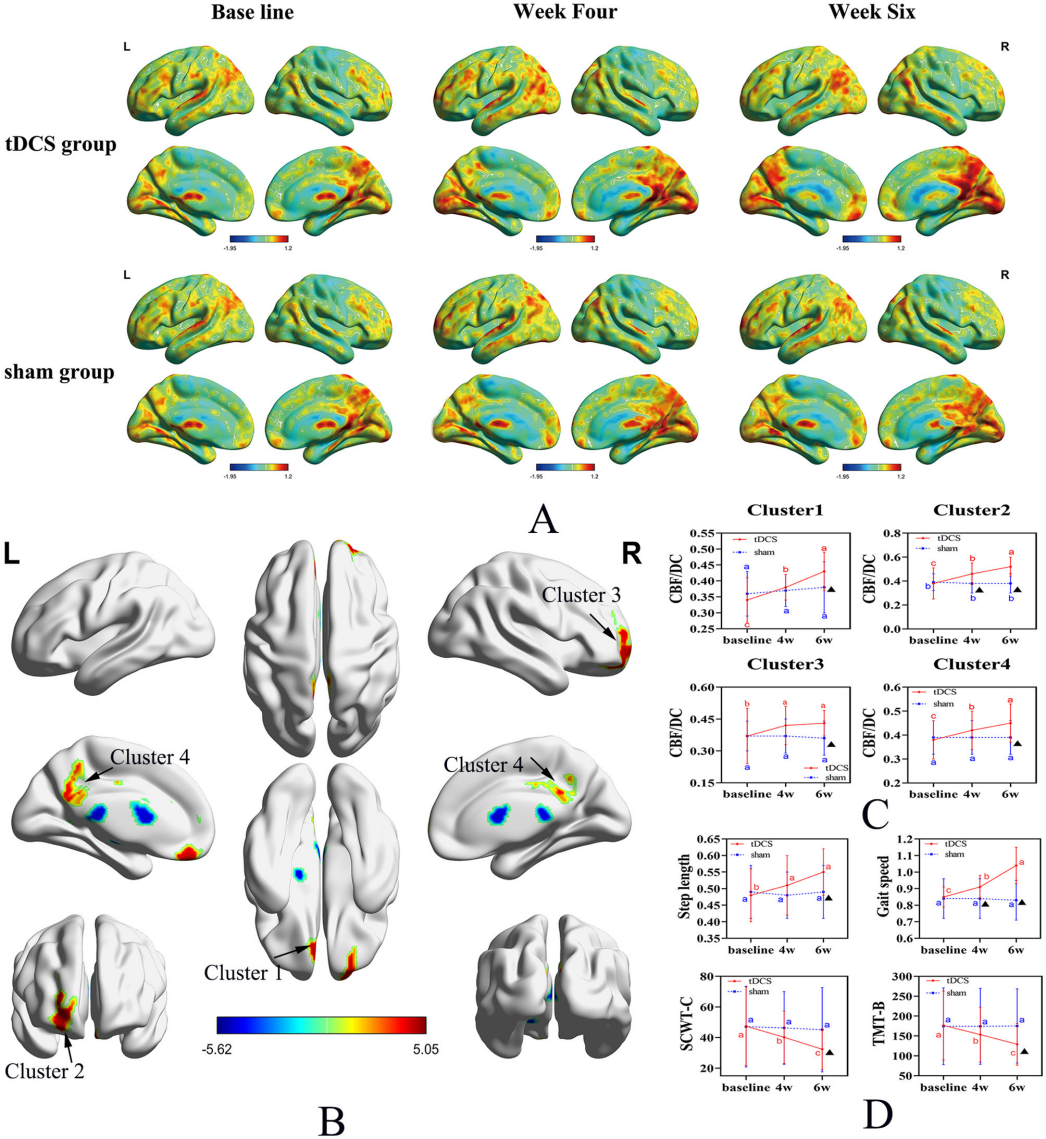
Spatial distribution maps of CBF/DC changes in the tDCS and Sham groups across three-time points. **(A)** Spatial distribution maps of average CBF/DC at baseline and the fourth and sixth weeks for both groups, with DC and CBF values normalized to *z*-scores and averaged within each group. The DC values were calculated for connectivity using a threshold of 0.25. **(B)** Brain regions showing differences in CBF/DC values between the tDCS and Sham groups. **(C)** Changes in CBF/DC values in four distinct clusters. **(D)** Changes in secondary outcome measures. Uppercase letters represent the time point differences shown in the letter labeling method (within-group comparison), and the asterisks indicate significant differences compared to the control group (within-group). CBF, cerebral blood flow; DC, degree centrality; tDCS, transcranial direct current stimulation; SCWT, Stroop Color-Word Test; TMT, Trail Making Test.

Repeated measures ANOVA was conducted for three time periods (baseline, week 4, and week 6) in both groups, revealing significant increases in the CBF/DC ratios across four brain clusters in both groups. Cluster 1 included the left gyrus rectus (REC.L) and the left anterior cingulate and paracingulate gyri (ACG.L); Cluster 2 included the left orbital mid frontal gyrus (ROBmid.L) and the left orbital superior frontal gyrus (ROBsup.L); Cluster 3 was mainly composed of the right orbital superior frontal gyrus (ROBsup.R) and the right orbital mid frontal gyrus (ROBmid.R). Cluster 4 included the left median cingulate and paracingulate gyri (DCG.L), right median cingulate and paracingulate gyri (DCG.R), and the left posterior cingulate gyrus (PCG.L), and the left precuneus (PAUN.L) (*P* < 0.05, FDR correction; Figures 2A, B, Table 2). A significant interaction effect between time and group was observed in all four clusters [*F* = 9.175, 28.589, 3.955, and 14.367 (Clusters 1–4); *P* = 0.001, *P* < 0.001, *P* < 0.023, and *P* < 0.001, respectively; Figure 2C, Table 3].

**Table 2:** Coordinates of brain regions in the tDCS and Sham groups for positive and negative CBF/DC ratios.

**Group**	**Brain regions**	**Cluster size (voxel)**	**MNI coordinate**			***F*-values**
**tDCS and Sham groups**			***X***	***Y***	***Z***	
Cluster 1	REC.L	153	−4	44	−22	4.40
	ACG.L	120				
Cluster 2	ROBmid.L	98	−24	48	−14	4.49
	ROBsup.L	44				
Cluster 3	ROBsup.R	172	26	60	−12	4.66
	ROBmid.R	126				
Cluster 4	DCG.L	323	0	−42	28	5.05
	DCG.R	276				
	PCG.L	202				
	PAUN.L	129				

tDCS, transcranial direct current stimulation. REC.L, the left gyrus rectus; ACG.L, left anterior cingulate and paracingulate gyri; ROBmid.L, left orbital mid frontal gyrus; ROBsup.L, left orbital superior frontal gyrus; ROBsup.R, right orbital superior frontal gyrus; ROBmid.R, right orbital mid frontal gyrus; DCG.L, left median cingulate and paracingulate gyri; DCG.R, right median cingulate and paracingulate gyri; PCG.L, left posterior cingulate gyrus; PAUN.L, left precuneus; CBF/DC, cerebral blood flow/degree centrality ratio; MNI, Montreal Neurological Institute.

**Table 3:** Primary and secondary outcomes.

	**tDCS group vs. Sham group Mean (SD)**			
	**tDCS group (*****n*** = **31)**	**Sham group (*****n*** = **29)**	**95% CI**	***P*** **-value**
**Primary outcomes**				
**Difference block CBF/DC value at week 6**				
Cluster 1	0.43 (0.06)	0.38 (0.08)	0.05 (0.01–0.09)	0.009
Cluster 2	0.52 (0.08)	0.38 (0.08)	0.14 (0.09–0.18)	< 0.001
Cluster 3	0.43 (0.06)	0.36 (0.08)	0.07 (0.02–0.11)	0.003
Cluster 4	0.45 (0.08)	0.39 (0.07)	0.06 (0.02–0.10)	0.009
**Secondary outcomes**				
**Gait and cognitive scales changed at week 6**				
Step length (m)	0.55 (0.07)	0.49 (0.08)	0.07 (0.02 to 0.11)	0.003
Gait speed (m/s)	1.05 (0.11)	0.83 (0.12)	0.22 (0.15 to 0.28)	< 0.001
SCWT-C (s)	32.34 (13.32)	45.10 (27.52)	−12.763 (−25.17 to −0.359)	0.044
TMT-B (s)	129.00 (52.65)	175.21 (93.27)	−46.22 (−89.77 to −2.67)	0.038

tDCS, transcranial direct current stimulation; SCWT, Stroop Color-Word Test; TMT, Trail Making Test; CBF/DC, cerebral blood flow/degree centrality ratio; SD, standard deviation; CI, confidence interval.

Multiple comparisons indicated that, in the tDCS group, CBF/DC values in Clusters 1, 2, and 4 significantly increased at baseline in Week 4 and Week 6 (*P* < 0.001). In Cluster 3, CBF/DC values were significantly higher at Weeks 4 and 6 than at baseline (*P* = 0.022 and *P* = 0.004, respectively), but not between the 4th and 6th weeks (*P* = 0.828). In the Sham group, there were no significant differences in CBF/DC values in the four regions across the three time points (*P* > 0.05; Figure 2C, Table 3).

### 3.3 Secondary outcomes

The tDCS group exhibited significant improvements in step length and walking speed compared to the Sham group, with a significant time × group interaction (*F* = 6.67 and 46.56; *P* = 0.003 and *P* < 0.001, respectively; Figure 2D, Table 3), and in processing abilities (SCWT-C and TMT-B tests), with a significant time × group interaction (*F* = 4.02 and 11.57; *P* = 0.025 and *P* < 0.001, respectively; Figure 2D, Table 3).

### 3.4 Correlation analysis of regions of interest

**Figure 3:**
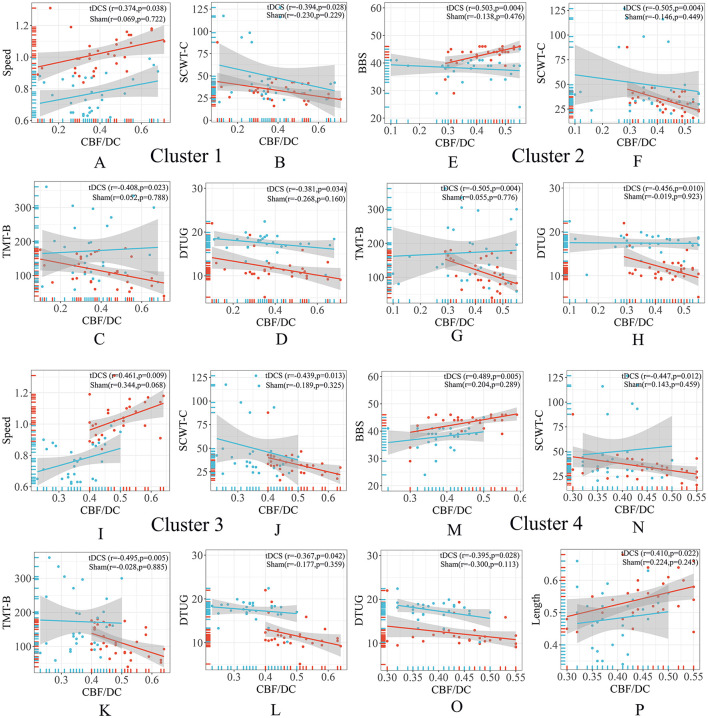
Correlation analysis of ROIs. **(A–D)** The CBF/DC ratio in the Cluster 1 region was correlated with gait characteristics and neuropsychological scales. **(E–H)** The CBF/DC ratio in the Cluster 2 region was correlated with gait characteristics and neuropsychological scales. **(I–L)** The CBF/DC ratio in the Cluster 3 region was correlated with gait characteristics and neuropsychological scales. **(M–P)** The CBF/DC ratio in the Cluster 4 region was correlated with gait characteristics and neuropsychological scales. BBS, Berg Balance Scale; CBF, cerebral blood flow; DC, degree centrality; DTUG, dual-task Timed Up and Go test; ROIs, regions of interest; SCWT, Stroop Color-Word Test; TMT, Trail Making Test. The warm colors represent the tDCS group, while the cool colors represent the sham group.

The CBF/DC ratio in the Cluster 1 region was correlated positively with walking speed and negatively correlated with SCWT-C, TMT-B, and DTUG scores (Figures 3A–D). In the Cluster 2 region, the CBF/DC ratio was correlated positively with walking BBS scores and negatively correlated with SCWT-C, TMT-B, and DTUG scores (Figures 3E–H). In the Cluster 3 region, the CBF/DC ratio was positively correlated with walking speed and negatively correlated with SCWT-C, TMT-B, and DTUG scores (Figures 3I–L). In the Cluster 4 region, the CBF/DC ratio was positively correlated with BBS scores and step length and negatively correlated with SCWT-C and DTUG scores (Figures 3M–P).

### 3.5 Adverse effects and safety

The incidence of adverse reactions was similar in the two groups (Table 4). No severe adverse reactions, such as hospitalization, suicide attempts, or seizures, were reported.

**Table 4:** Frequency of adverse effects.

**Adverse effect**	**tDCS group (*N* = 33)**	**Sham group (*N* = 33)**	***P*-value**
**Week 1**			
Headache, *n* (%)	3 (2.5)	2 (2.5)	>0.99^a^
Neck pain, *n* (%)	2 (2.0)	2 (2.0)	>0.99^a^
Burning sensation, *n* (%)	8 (5.0)	2 (5.0)	0.039^a^
Scalp pain, *n* (%)	4 (3.5)	3 (3.5)	>0.99^a^
Tinnitus, *n* (%)	3 (2.0)	1 (2.0)	0.613^a^
Skin redness, *n* (%)	5 (3.5)	2 (3.5)	0.427^a^
Sleepiness, *n* (%)	4 (3.5)	3 (3.5)	>0.99^a^
Trouble concentrating, *n* (%)	3 (2.5)	2 (2.5)	>0.99^a^
**Week 4**			
Headache, *n* (%)	3 (2.5)	2 (2.5)	>0.99^a^
Neck pain, *n* (%)	3 (2.0)	1 (2.0)	0.613^a^
Burning sensation, *n* (%)	3 (2.5)	2 (2.5)	>0.99^a^
Scalp pain, *n* (%)	2 (1.5)	1 (1.5)	>0.99^a^
Tinnitus, *n* (%)	3 (2.5)	2 (2.5)	>0.99^a^
Skin redness, *n* (%)	4 (3.5)	3 (3.5)	>0.99^a^
Sleepiness, *n* (%)	2 (2.0)	2 (2.0)	>0.99^a^
Trouble concentrating, *n* (%)	3 (2.5)	2 (2.5)	>0.99^a^
**Week 6**			
Headache, *n* (%)	1 (1.0)	1 (1.0)	>0.99^a^
Neck pain, *n* (%)	2 (1.5)	1 (1.5)	>0.99^a^
Burning sensation, *n* (%)	2 (1.5)	1 (1.5)	>0.99^a^
Scalp pain, *n* (%)	1 (1.0)	1 (1.0)	>0.99^a^
Tinnitus, *n* (%)	0 (0.5)	1 (0.5)	>0.99^a^
Skin redness, *n* (%)	2 (1.5)	1 (1.5)	>0.99^a^
Sleepiness, *n* (%)	2 (1.5)	1 (1.5)	>0.99^a^
Trouble concentrating, *n* (%)	2 (1.5)	1 (1.5)	>0.99^a^

^a^Fisher exact test.

tDCS, transcranial direct current stimulation.

## 4 Discussion

### 4.1 Main findings

In this study, we investigated the clinical effects and safety of tDCS on brain perfusion, functional connectivity, gait characteristics, and cognitive executive function in patients with gait disorders associated with CSVD. Notably, the results were consistent with our hypothesis. The 6-week tDCS treatment improved whole-brain network connectivity in patients with CSVD. The CBF/DC ratio increased in four clusters, including the orbitofrontal cortex regions (OFC), comprising the REC.L, ROBmid.L, ROBsup.L, ROBsup.R, and ROBmid.R. Moreover, increased CBF/DC ratios were observed in the cingulate gyrus clusters, consisting of the left ACG.L, DCG.L, DCG.R, PCG.L, and PAUN.L. No significant adverse reactions were reported during the treatment period.

With advancements in neuroimaging, the role of the orbitofrontal cortex in cognition, risk decision-making, and emotional regulation has gained increasing support (Wallis, 2007; Zhuang et al., 2021; Hogeveen et al., 2022). The orbitofrontal region plays a crucial role in individual decision-making and performs normally in cognitive tests, supporting the mainstream view of OFC functionality (Knudsen and Wallis, 2022). Although previous studies have shown limited connections between the OFC and motor functions compared to other frontal lobe regions, limited studies have focused on its role in regulating body movements and posture balance (Padoa-Schioppa and Assad, 2006; Kennerley et al., 2009). This limited connection is consistent with its anatomical structure, which exhibits stronger connections with autonomic control regions than with muscle-skeletal control regions (Cavada et al., 2000). However, with the concept of cognitive mapping, the OFC has been recognized for its complex and crucial role. While it was initially believed that neural instantiation of cognitive maps mainly occurs in the hippocampus, neuroimaging findings indicate that when individuals utilize cognitive maps, the OFC is the only cortical region activated, suggesting its significant role (Wikenheiser and Schoenbaum, 2016). Therefore, any sufficiently complex task, including physical activities such as movement, should benefit from cognitive mapping (Zhuang et al., 2021). The execution of complex movements requires advanced cognitive functions to provide feedback on various sensory transmissions and environmental controls (Scherder et al., 2007; Annweiler et al., 2013; Collyer et al., 2022). Current research has confirmed a significant correlation between cognitive levels and gait characteristics, with cognitive improvement contributing to enhanced gait features (Scherder et al., 2007; Annweiler et al., 2013; Cohen et al., 2016). Notably, this correlation is consistent with our finding of increased CBF/DC values in the bilateral OFC regions following treatment with tDCS, leading to significant improvements in gait characteristics and executive processing abilities.

As previously mentioned, we observed increased CBF/DC values in the left cingulate gyrus and DCG.R regions. The cingulate gyrus is a cortical part of the limbic system, with the ACG being part of the attention network and projecting to the spinal cord. It closely interacts with the lateral PFC, thalamus, pre-motor, and supplementary motor areas, regulating attention or executive function, complex motor control, motivation, error monitoring, and working memory by influencing sensory feedback or response selection (Brockett et al., 2020; Bubb et al., 2021). Enhancing neurovascular coupling in this area contributes to improved executive and motor functions. Notably, visual perception enables a unified understanding of external stimuli. The initial input information is broken down into features, such as shape, color, and motion, which are processed in different brain regions and then integrated to form a unified cognitive representation of the object, governing the regulation of postural movement. The PAUN region plays a crucial intermediary role in this process. Furthermore, the ACG is associated with various high-level cognitive functions, such as episodic memory, self-relevant information processing, coordinating motor behavior, and guiding attention toward relevant information. It is also part of the default mode network, contributing to external supervision and visual-spatial attention (Li et al., 2019; Dadario and Sughrue, 2023; Jitsuishi and Yamaguchi, 2023). The increased CBF/DC values in the PAUN region observed in our study following tDCS suggest enhanced patient attention, thereby improving postural movement regulation.

### 4.2 Secondary outcomes

In the tDCS group, we observed significant improvements in CBF and functional connectivity. However, deep nuclei, such as the thalamus and caudate nucleus, exhibited no significant differences compared to the Sham group. We speculate that long-term tDCS increases CBF flow and induces concurrent changes in synaptic plasticity, which may contribute to improved CSVD-GD motor performance through increased CBF involved in motor regulation and synaptic plasticity changes in the basal ganglia. Furthermore, tDCS may enhance synaptic transmission through long-term stimulation, affecting communication between neurons.

Treatment with tDCS significantly improved gait characteristics and cognitive executive function in patients with CSVD despite no significant changes in overall cognitive levels (MoCA scores). These improvements were evidenced by noticeable increases in stride length and walking speed, as well as decreased scores in tasks such as TMT and SCWT. These changes in gait parameters may be related to tDCS-induced modulation of various brain regions involved in coordinating walking movements, including the fronto-temporal-striatal network and the basal ganglia (Nishida et al., 2024).

### 4.3 Clinical and research implications

Effective treatment methods for patients with CSVD-GD are currently lacking, and existing research results are inconsistent (Yang et al., 2021; Veldema and Gharabaghi, 2022; Shibata et al., 2023). Our study highlights the clinical significance of tDCS, demonstrating that long-term, repeated tDCS therapy significantly improves gait and cognitive function in patients with CSVD-GD. These findings provide a simple, reliable, and non-invasive physical therapy option for neurodegenerative diseases.

After the tDCS intervention, we analyzed changes in multiple MRI parameters and cognitive behavioral scale scores in patients with CSVD-GD. We identified early characteristic changes, such as increased neurovascular coupling in the orbitofrontal cortex, cingulate gyrus, and the PAUN, along with improvements in executive function and gait. These findings provide a theoretical basis for early interventions. The findings emphasize the potential of tDCS as a non-invasive intervention, highlighting its role in early intervention strategies and suggesting directions for future research and clinical applications. No serious adverse events were reported in our study, and the safety profile further supports the clinical applicability of tDCS, consistent with earlier findings (Ko, 2021), reinforcing tDCS as a safe and effective intervention for various neurological disorders.

### 4.4 Limitations

This study has some limitations. First, the results are based on a small sample size; therefore, future research should include a larger sample sizes. Second, this study did not incorporate more comprehensive reports based on whole-brain voxel-based morphometry and blood biomarkers.

In conclusion, the present study demonstrated the effects of tDCS by monitoring changes in neural connectivity and CBF coupling using MRI. With extended observation periods and proper risk assessment, tDCS can serve as an additional intervention for treating CSVD-GD. In the future, we aim to delve deeper into the effects of tDCS on CSVD-GD over longer durations, different regions, and varying stimulation intensities. Future research requires larger sample sizes and comprehensive analyses of changes in multimodal imaging of patients with CSVD-GD to validate these findings further.

## Data Availability

The original contributions presented in the study are included in the article/Supplementary material, further inquiries can be directed to the corresponding authors.
