# Supplementary material for: Transcranial direct current stimulation for patients with walking difficulties caused by cerebral small vessel disease: a randomized controlled study
Source: Front Aging Neurosci. 2025 Jan 7;16:1511287. doi: 10.3389/fnagi.2024.1511287 (PMC11756518; doi:10.3389/fnagi.2024.1511287)
Supplement: Supplementary file 1 [file Data_Sheet_1.pdf]

Supplemental materials

Experimental Design

To ensure the randomness of the study and reduce potential selection bias, we implemented a rigorous randomization method during the participant recruitment process. The specific steps are as follows:

Participant Screening: All participants underwent a strict screening process before entering the study to ensure they met the inclusion and exclusion criteria. **Randomization Method:** Participants were allocated using computer-generated random numbers. Specifically, we input the names of all eligible participants into randomization software, which automatically generated random numbers. Participants were then assigned sequentially to the experimental group (tDCS intervention group) or the control group (sham stimulation group) based on the random order produced. **Grouping Ratio:** In our design, participants were allocated to the experimental group or the control group in a 1:1 ratio.

Blind Design: Throughout the trial, both participants and evaluators were unaware of their group assignments. The blind design of the assessment outcomes ensured that potential biases during the evaluation process were minimized. Only the study coordinator and the researchers responsible for administering the stimulation were aware of the group allocations. By providing a detailed description of the randomization process, we ensure that future researchers can understand and replicate this procedure, thereby enhancing the transparency of the randomization.

Evaluation of the Success Rate of Blinding: To assess the success rate of the

single-blind method, our team asked the evaluators during the final follow-up whether they had guessed the group assignment of the participants. If the evaluators were unable to accurately identify the group assignment, it indicated that the blinding was successful. Among the sham stimulation group, five patients expressed doubts about the effectiveness and intensity changes of the stimulation but were not explicitly aware of their group assignment.

MRI scanning and processing

Scans were performed using the 3.0T Discovery MR750w MRI system (GE Healthcare, USA), equipped with a 24-channel head coil. Participants were instructed to lie quietly with their eyes closed, avoiding head movement, falling asleep, or engaging in cognitive tasks (i.e., thinking of their problems. The scanning sequences included 3D pseudo-continuous arterial spin labeling (3D-pCASL), three-dimensional brain volume T1-weighted imaging (3D-BRAVO T1WI), T2-weighted fluid-attenuated inversion recovery (T2WI FLAIR), and susceptibility weighted imaging (SWI).

The parameters for CBF scanning were as follows: TR = 5070 ms, TE = 11.48 ms, post-labeling delay time = 2,000 ms, flip angle = 111°, matrix size = 128×128, FOV = 240 mm×240 mm, slice thickness = 3 mm, number of slices = 50, The total acquisition time was 4 min 4s

(2) rs-fMRI scanning: SE-EPI sequence imaging. TR = 2 ms, TE = 30 ms, FOV = 240 × 240 mm, flip angle = 90°, matrix size = 64 × 64, slice thickness = 4 mm, and slice gap = 0.6 mm.

CBF data preprocessing and computation process

The CBF data preprocessing comprised the following stages: (1) Checking data quality and converting image format to NIFTI format; (2) Non-linearly registering the cerebral blood flow images to the positron emission tomography image template provided by the Montreal Neurological Institute (MNI) and performing image quality check; (3) Data normalization: z-score transformation of cerebral blood flow values for each voxel by dividing the value by the mean cerebral blood flow value of the whole brain; (4) Performing Gaussian smoothing with a full width at half maximum of 6 mm; (5) Correcting multiple comparisons using the false discovery rate (FDR) method and a threshold of $P < 0.05$; (6) Non-linearly transforming the results to the anatomical automatic labeling (AAL) template, and presenting the final results using xjview (Beijing, CN) at https://www.alivelearn.net/xjview/ (accessed on 12 August 2023) and BrainNet Viewer (Beijing, CN) at http://www.nitrc.org/projects/bnv/ (accessed on 12 August 2023) software.

fMRI data preprocessing and computation process

Data preprocessing was performed using Statistical Parametric Mapping 12 (SPM12; http://www.fil.ion.ucl.ac.uk/spm) on the MATLAB platform. The resting-state fMRI data processing assistant software (DPARSF, http://rfmri.org/dparsf; DPABI 4.3, http://rfmri.org/dpabi) was utilized for data preprocessing. Each participant’s first 10 volumes of functional data were discarded, following (http://rfmri.org/dparsf

(accessed on 12 August 2023)) temporal and head motion correction. Subsequently, all images were normalized to the standard Montreal Neurological Institute template and resampled to a resolution of 3 × 3 × 3 mm. A set of 24 head motion parameters and average signals from the white matter, cerebrospinal fluid, and global signals were employed as nuisance covariates to minimize the impact of head motion and non-neuronal BOLD fluctuations. Finally, the detrending process was applied to eliminate the offset caused by the sensor during data processing. Subsequently, the time series of each voxel was extracted, and the Pearson correlation coefficient with all other voxels in the brain, using a threshold set at 0.25, was calculated. A lower threshold may include false positive connections, while a higher threshold may exclude some meaningful connections. Consequently, the correlation coefficients were transformed using Fisher-Z transformation to improve normality.

Authorization certificate for the Berg Balance Scale (BBS)

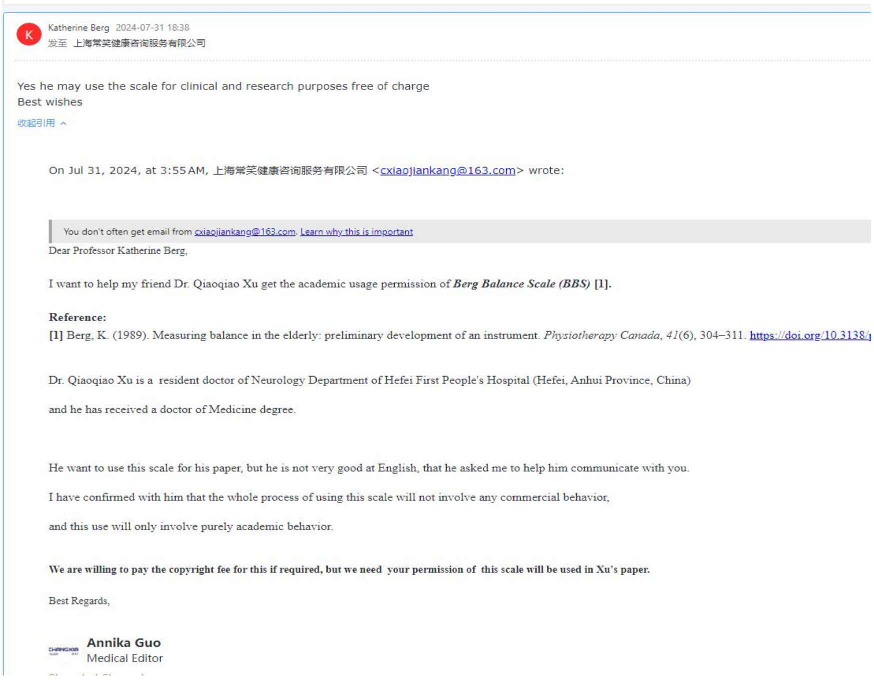
K Katherine Berg 2024-07-31 18:38
发至 上海常笑健康咨询服务有限公司

Yes he may use the scale for clinical and research purposes free of charge
Best wishes
收起引用

On Jul 31, 2024, at 3:55 AM, 上海常笑健康咨询服务有限公司 <cxiaojiankang@163.com> wrote:

You don't often get email from cxiaojiankang@163.com. Learn why this is important

Dear Professor Katherine Berg,

I want to help my friend Dr. Qiaoqiao Xu get the academic usage permission of ***Berg Balance Scale (BBS)*** **[1].**

Reference:
[1] Berg, K. (1989). Measuring balance in the elderly: preliminary development of an instrument. *Physiotherapy Canada*, *41*(6), 304–311. https://doi.org/10.3138/[illegible]

Dr. Qiaoqiao Xu is a resident doctor of Neurology Department of Hefei First People's Hospital (Hefei, Anhui Province, China)

and he has received a doctor of Medicine degree.

He want to use this scale for his paper, but he is not very good at English, that he asked me to help him communicate with you.

I have confirmed with him that the whole process of using this scale will not involve any commercial behavior,

and this use will only involve purely academic behavior.

We are willing to pay the copyright fee for this if required, but we need your permission of this scale will be used in Xu's paper.

Best Regards,

Annika Guo
Medical Editor

Authorization certificate for the TUG test.

Licensed Content Date	Apr 27, 2015
Licensed Content Volume	39
Licensed Content Issue	2
Licensed Content Pages	7
Type of use	Dissertation/Thesis
Requestor type	University/Academic
Format	Electronic
Portion	Text extract
Number of Pages	1
Will you be translating?	No
Title of new work	Long-Term Impact of Transcranial Direct Current Stimulation in Patients with Walking Difficulties Caused by Cerebral Small Vessel Disease

JOHN WILEY AND SONS LICENSE
TERMS AND CONDITIONS

Aug 01, 2024

This Agreement between Qiaoqiao Xu ("You") and John Wiley and Sons ("John Wiley and Sons") consists of your license details and the terms and conditions provided by John Wiley and Sons and Copyright Clearance Center.

License Number	5840091302822
License date	Aug 01, 2024
Licensed Content Publisher	John Wiley and Sons
Licensed Content Publication	Journal of the American Geriatrics Society
Licensed Content Title	The Timed "Up & Go": A Test of Basic Functional Mobility for Frail Elderly Persons
Licensed Content Author	Sandra Richardson, Diane Podsiadlo

Institution name	Hefei First People's Hospital
Expected presentation date	Sep 2024
Order reference number	MR-34-24-022386
Portions	page 2
The Requesting Person / Organization to Appear on the License	Qiaoqiao Xu
Requestor Location	Hefei First People's Hospital<br>No.390 Huaihe Road<br>Luyang District, Hefei City<br>Anhui Province<br>Hefei, Anhui 230071<br>China<br>Attn: Hefei First People's Hospital
Publisher Tax ID	EU826007151
Total	0.00 USD
